# Characteristics and chronologically changing patterns of late-onset breast cancer in Korean women of age ≥ 70 years: A hospital based-registry study

**DOI:** 10.1186/s12885-022-10295-y

**Published:** 2022-12-05

**Authors:** Hyun-June Paik, Suk Jung Kim, Ku Sang Kim, Yongsuk Kim, Se Kyung Lee, Su Hwan Kang, Jeong Joon, Hyun Jo Youn, Korean Breast Cancer Society

**Affiliations:** 1 Department of Surgery, Pusan National University Yangsan Hospital, Yangsan, South Korea; 2 Department of Radiology, Haeundae Paik Hospital, Inje University College of Medicine, Haeundae-ro 875, Haeundae-gu, Busan, 612-030 South Korea; 3 Department of Breast-Endocrine Surgery, Kosin University Gospel Hospital, Busan, South Korea; 4 Department of Surgery, Uijeongbu St. Mary’s Hospital, The Catholic University of Korea, Uijeongbu, South Korea; 5 Division of Breast and Endocrine Surgery, Department of Surgery, Samsung Medical Center, Seoul, South Korea; 6 Department of Surgery, Yeungnam University College of Medicine, Daegu, South Korea; 7 Department of Surgery, College of Medicine, Yonsei University, Seoul, South Korea; 8 Department of Surgery, Chonbuk National University Medical School, Jeonju, South Korea

**Keywords:** Old-onset breast cancer, Triple-negative breast cancer, Asia, Korea

## Abstract

**Background:**

Women from Asian and western countries have vastly different ages of onset of breast cancer, with the disease tending to occur at an older age in the West. Through an investigation of the patterns of old-onset breast cancer (OBC) in Korean women, we aimed to identify the characteristics of Korean OBC and evaluate whether these patterns are changing in relation to increasing westernization.

**Methods:**

This study retrospectively evaluated 102,379 patients who underwent surgical treatment of primary breast cancer between January 1, 2000 and December 31, 2013 in Korea. We used hospital -based breast cancer registry and analyzed data from these patients using multiple linear regression analysis to compare the characteristics and chronologically changing patterns between OBC (70 years of age or older) and non-OBC (40–69 years of age) patients in Korea.

**Results:**

A total of 6% of the 102,379 patients had OBC. Overall, OBC had more favorable biological features, such as a higher incidence of luminal A subtype, than did non-OBC, except for a higher incidence rate of triple-negative breast cancer (TNBC). However, OBC also presented with a higher overall disease stage, including higher T and M stages. Although the incidence rates of both OBC and non-OBC have increased overtime, the relative proportion of OBC patients has slightly increased, whereas that of non-OBC has slightly decreased. The increase in the incidence of both OBC and non-OBC was primarily due to the luminal A subtype.

**Conclusions:**

Based on a hospital-based registry, overall, Korean OBC had favorable biological features but showed a higher rate of TNBC and advanced cancer stages. The incidence trend of breast cancer in Korea is slowly shifting toward an older age at onset, largely due to the luminal A subtype. Our results may provide novel insights into OBC in Asia, and aid in the development of optimal management of the disease in Asia.

**Trial registration:**

Retrospectively registered.

## Background

Currenly, breast cancer is the most prevalent among other famale malignancies globally [1, 2]. Because the incidence of breast cancer and the associated mortality have been shown to increase with age in developed countries, aging is recognized as the strongest risk factor for the disease [3]. Although the incidence and mortality associated with breast cancer have been decreasing in developed countries since the early 2000s, this trend is less significant or even reversed among elderly women [4]. In 2012, breast cancer in women aged 70 years or older accounted for 30.1% of the entire new breast cancer diagnoses and 51.2% of breast cancer mortalities in developed countries [3]. Moreover, with the global population trend in aging, late-onset breast cancer diagnosis poses a significant disease burden. More attention is now directed towards the epidemiology, clinical and biological aspects, and clinical results of old-onset breast cancer (OBC) than of non-OBC [3, 5].

Regarding breast cancer, Asia faces more rapid increases in the incidence rate than does the West [6]. In 2020, Asians accounted for, 1,026,171 (45.4%) of the 2,261,419 new diagnoses of breast cancer worldwide [7]. Asian and western populations are starkly different in terms of their breast cancer-related epidemiologic and biologic profiles [8–10]. Noticeably, the peak age at disease onset is much younger in Asia than in the West (40–50 years vs. 60–70 years) [8]. Despite the lower incidence rate in the older age group in Korean women, the incidence rate increased mostly in the oldest group, age ≥ 70 years. It was reported as 2.3 times among women in their thirties, 2.5 times among those in their forties, 2.6 times among women in their fifties, 3.4 times among those in their sixties, and 3.8 times among women in their seventies, between 1998 and 2010 [11]. Nevertheless, most of the global research studies on breast cancer in elderly women have focused on western populations, and there is a relative lack of relevant data for Asian populations [3].

We intended to assess the features of breast cancer in elderly Korean women and determine the variations in the incidence and subtypes of OBC over time. Finally, we also intended to evaluate the future trends in OBC in Korea.

## Methods

### Korean Breast Cancer Registry

The entire dataset on breast cancer cases in this study were derived from the Korean Breast Cancer Registry (KBCR). The KBCR is a multiinstituional, online breast cancer database collected in a prospective manner (http://registry.kbcs.or.kr/ecrf/login.php), which was estalished by the Korean Breast Cancer Society (KBCS) 〔11〕. More than100 instituions with over 400 beds each volountarilly took part in this registry 〔11〕. In 2013, it was assumed that this registry would reflect over 65% of new breast cancer cases detected in Korea [12]. The KBCR provides diverse data on breast cancer research, beyond basic registry data (sex, age), encompassing social circumstances (region, education level, marital status), clinical information (body mass index, history of breast feeding, menopausal status), pathological results (histological features, stage in accoradence with the American Joint Committee on Cancer classification, biomarker expressions of estrogen receptors [ERs], progesterone receptors [PRs], human epidermal growth factor receptor 2 [HER2]), and treatment methods (surgery, neoadjuvant, adjuvant therapy) 〔11〕.

By contrast, the Korean Central Cancer Registry (KCCR) is a nationwide cancer database established by the Korean Ministry of Health and Welfare, and the KCCR data includes the entire cancer cases in Korea (https://kccrsurvey.cancer.go.kr/index.do). However, the KCCR only contains cancer data confined to general epidemiology (incidence, mortality, survival rate), brief patient information (age, sex, and region) .

### Definition of OBC and breast cancer incidence

Previous studies on breast cancer in older women show inconsistencies in the definition of the OBC cutoff age [3, 13–16]. We defined OBC as breast cancer developing in a woman aged 70 years or older. We further defined breast cancer incidenace as the number of newly detected breast cancer case per 100 Korean women per year 〔17〕. The exclusion criteria were breast cancer cases without information on cancer stage or surgical methods used. Therefore, the incidence of breast cancer, as reported in our study, is not equal to the whole breast cancer incidence in Korea recorded by the nation-wide registry data.

### Definition of breast cancer subtypes

In respect of molecular subtype classification of breast cancer, immunohistochemical surrogates for ER, PR, HER2 status were used to determine breast cancer subtypes. Positivity or negetivity of ER and PR expression was solely based on immunohistochemistry (IHC) staining, whereas positivity or negetivity of HER2 expression was based on both IHC staining and in situ hybridization (fluorescence [FISH] or silver [SISH] in situ hybridization methods). IHC reults of zero or 1+ (week) HER2 staining were considered to have a negative HER2 status, whereas those with 3 + (strong) HER2 staining were considered to have a positive. For cases with 2+ (equivocal) HER2 staining, FISH or SISH results that determined the the final HER2 expression; cases with positive FISH or SISH results were considered as HER2-positive, whereas those with negative FISH or SISH results were considered as HER2-negative. Cases with missing FISH or SISH results were determined as having an unknown HER2 status.

According to the status of hormone receptor and HER2 expression, patients were classified into four breast cancer subtypes. These subtypes included ‘luminal A’: postive-ER and/or PR and negative-HER2; ‘luminal B’: positive-ER and/or PR and positive- HER2; ‘triple negative breast cancer (TNBC)’: negative - ER and PR and negative -HER2; ‘HER2’: negative-ER and PR and postive–HER2.

### Study population (Fig. 1)

This study attained an approval from the Instituional Review Board of Haeundae Paik Hospital (Institutional Review Board file no. 2019–09-010). The KBCS anonymized and de-identified personal information from the records for the protection of patient privacy.

**Fig. 1:**
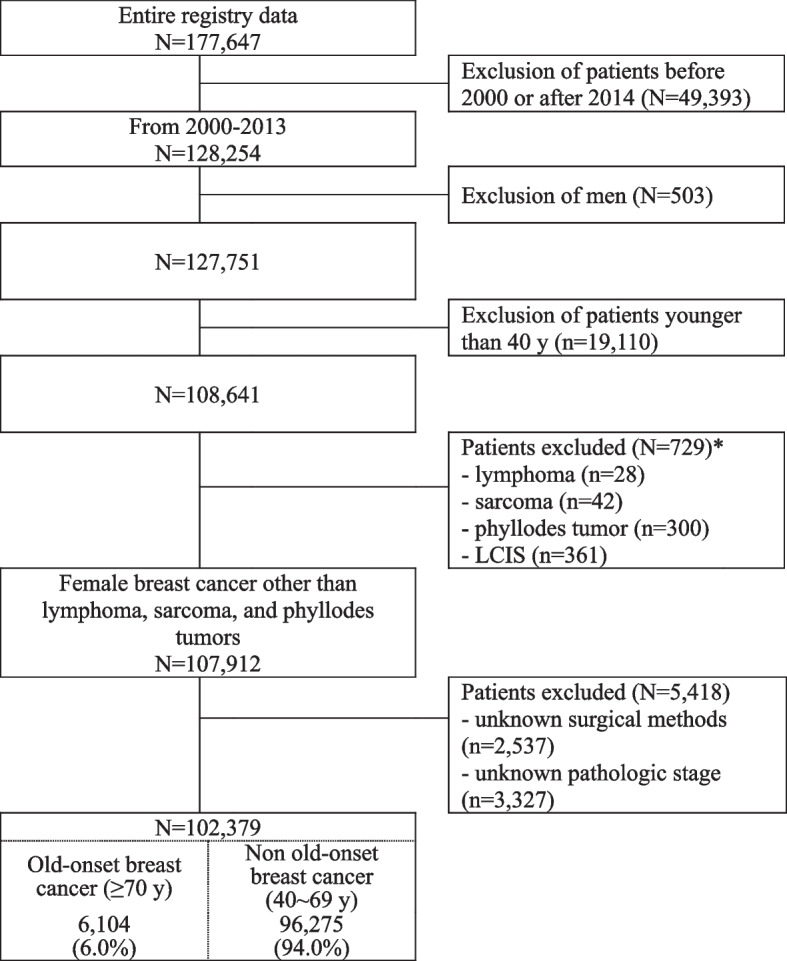
Patient selection. LCIS, lobular carcinoma in situ

For this analysis, we excluded male patients, patients aged less than 40 years, those with a histopathology of lobular carcinoma in situ, phyllodes tumor, sarcoma and lymphoma, cases without data on age, types of surgery, pathological stages, and those diagnosed prior to the year 2000 owing to their questionable reliability. Patients younger than 40 years of age were excluded from the non-OBC group because young-age breast cancer (under 40 years of age) is reported to have dissimilar clinicopathological characteristics compared with older-age breast cancer (age 40 years and older) [17]. In addition, the issue of patients aged less than 40 years had already been intensively dealt and analyzed in the preceding study which used the identical database and study period as this study [17]. In this retrospective study drawn from a hostpial-based Korean registry, we finally included 102,379 patients who underwent surgical treatment of primary breast cancer from January 1, 2000 to December 31, 2013 in Korea.

Based on this dataset, we compared between the OBC and non-OBC groups to assess the features of OBC and examine chronological changes in incidence rates of OBC. To assess chronological changes in subtypes, we performed subset analysis of 76,349 cases having accurate data on status of ER, PR, and HER2 expression.

### Statistical analysis

Regarding comparison between the OBC and non–OBC groups, clinicopathological features presented as categorial variables were compared using Fisher’s exact or Chi-squared test. However, those presented as continous variables were compared using Mann-Whitney U or independent test. A large amount of data on clinicopathological variables were missing, and the missing data were excluded from the analysis. To check the normality of the distribution, we used the Shapiro-Wilk test. To compare changes in the frequencies, incidences, and proportions with time among different age and subtype groups, we performed multiple linear regression test and checked interaction effects between variables regarding changes. Bonferroni-corrected *p* values were calculated for multiple comparison tests. Estimates of the frequency and incidence of each subtype were derived by the slope of the regression line, which represents the annual rate of change in the frequency and incidence. For all statistical analyses, SPSS version 24.0 was used, and *p* values less than 0.05 were idenified as having statistial significance.

## Results

### Clinicopathological features of OBC in Korea

Of a total of 102,379 patients, 6.0% had OBC. A comparison of the OBC and non-OBC data is shown in Tables 1 and 2. OBC was diagnosed at the mean age of 73 (range, 70–108) years. Patients with OBC were significantly more likely to have late menarche, late menopause, an early first delivery, high parity, lactation experience, be obese, have a lower education level, have undergone fewer previous mammographic screenings, a more symptomatic initial presentation including palpation of the tumor, undergone a total mastectomy, as well as lower rates of breast reconstruction, chemotherapy, and radiation therapy. OBC tumors showed favorable biologic features, except for a higher proportion of TNBC. Those favorable biologic features included a lower histologic grade, weaker lymphovascular invasion, higher negative HER2 status, a higher proportion of the luminal A subtype, and lower proportions of the luminal B and HER2 subtypes. However, OBC was associated with aggressive clinical manifestations related to symptomatic presentation, degree of palpation of the tumor, degree of negative PR expression, and advanced pathologic stage (advanced T, M, and overall stages).

**Table 1:** Clinical characteristics of elderly Korean women with breast cancer

		Group		
Variable	Total no. of analyzed patients (%)	Old-onset breast cancer (≥70)	Non old-onset breast cancer (40 ~ 69)	***p*** value
	(***N =*** 102,379)	(***N =*** 6104)	(***n =*** 96,275)	
**Age, years**	50 (40–108)	73 (70–108)	50 (40–69)	<.001^1^
	52.46 ± 9.09	74.21 ± 4.02	51.08 ± 7.41	
**Age at menarche, years**				
	20,574 (20.1)			<.001^2^
< 13	542	21 (0.9)	521 (2.8)	
13–16	13,410	1218 (55.0)	12,192 (66.4)	
≥ 17	6622	975 (44.0)	5647 (30.8)	
Missing	81,805	3890	77,915	
**Age at menopause onset, years**				
	20,574 (20.1)			<.001^2^
< 45	2277	229 (10.3)	2048 (11.2)	
45–54	16,084	1622 (73.3)	14,462 (78.8)	
≥ 55	2213	363 (16.4)	1850 (10.1)	
Missing	81,805	3890	77,915	
**Age at first delivery, years**				
	20,574 (20.1)			<.001^2^
< 23	4354	967 (43.7)	3387 (18.4)	
≥ 23	16,220	1247 (56.3)	14,973 (81.6)	
Missing	81,805	3890	77,915	
**Parity**	64,939 (63.4)			.911^2^
	64,939 (63.4)			<.001^2^
0	1967	116 (3.1)	1851 (3.0)	
1 ~ 2	45,244	884 (23.3)	44,360 (72.5)	
≥ 3	17,728	2792 (73.6)	14,936 (24.4)	
Missing	37,440	2312	35,128	
**BMI, kg/m**^**2**^				
	75,274 (73.5)			<.001^2^
< 18.5	1844	94 (2.1)	1750 (2.5)	
18.5–22.9	31,010	1146 (25.9)	29,864 (42.2)	
23–24.9	18,504	1003 (22.7)	17,501 (24.7)	
25–29.9	20,468	1821 (41.1)	18,647 (26.3)	
≥ 30	3448	363 (8.2)	3085 (4.4)	
Missing	27,105	1677	25,428	
**Family history**	73,757 (72.0)			.240^2^
Yes	5962	324 (7.6)	5638 (8.1)	
No	67,795	3935 (92.4)	63,860 (91.9)	
Missing	28,622	1845	26,777	
**Personal history**	102,379 (100.0)			.503^2^
Yes	2061	130 (2.1)	1931 (2.0)	
No	100,318	5974 (97.9)	94,344 (98.0)	
Missing	0	0	0	
**Lactation experience**	59,723 (58.3)			<.001^2^
Yes	43,705	3008 (88.7)	40,697 (72.2)	
No	16,018	383 (11.3)	15,635 (27.8)	
Missing	42,656	2713	39,943	
**Oral contraceptive use**	58,960 (57.6)			.493^2^
Yes	7009	384 (11.5)	6625 (11.9)	
No	51,951	2951 (88.5)	49,000 (88.1)	
Missing	43,419	2769	40,650	
**Hormone replacement therapy**	58,938 (57.6)			.142^2^
Yes	6415	340 (10.1)	6075 (10.9)	
No	52,523	3020 (89.9)	49,503 (89.1)	
Missing	43,441	2744	40,697	
**Education level**	49,715 (48.6)			<.001^2^
More than middle school	40,078	1077 (37.0)	39,001 (83.3)	
Less than middle school	9637	1830 (63.0)	7807 (16.7)	
Missing	52,664	3197	49,467	
**Prior mammography screening**	48,003 (46.9)			<.001^2^
Yes	27,876	1181 (43.4)	26,695 (59.0)	
No	20,127	1542 (56.6)	18,585 (41.0)	
Missing	54,376	3381	50,995	
**Presence of symptoms**	102,379 (100.0)			<.001^2^
Asymptomatic	21,404	1067 (17.5)	20,337 (21.1)	
Symptomatic	80,975	5037 (82.5)	75,938 (78.9)	
**Palpation of the tumor**	69,580 (68.0)			.009^2^
Yes	55,523	3321 (81.4)	52,202 (79.7)	
No	14,057	759 (18.6)	13,298 (20.3)	
Missing	32,799	2024	30,775	
**Breast surgery**	102,379 (100.0)			<.001^2^
Mastectomy	46,713	3798 (62.2)	42,915 (44.6)	
BCS	55,666	2306 (37.8)	53,360 (55.4)	
**Axilla surgery**	102,378(100.0)			<.001^2^
Sentinel lymph node biopsy	29,857	1899 (31.1)	27,958 (29.0)	
Axillary dissection	64,368	3669 (60.1)	60,699 (63.0)	
No surgery	8153	536 (8.8)	7617 (7.9)	
Missing	1	0	1	

Values are either frequency with percentage in parentheses or median (range)

^1^*P* values were derived from the Mann-Whitney U test

^2^ *P* values were derived from a Chi-square test

The Shapiro-Wilk test was employed to examine the normality assumption

*BMI* Body mass index

**Table 2:** Pathological and biological characteristics of elderly Korean women with breast cancer

		Group		
Variable	Total no. of analyzed patients (%)	Old-onset breast cancer (≥70)	Non old-onset breast cancer (40 ~ 69)	***p*** value
	(***N =*** 102,379)	(***N =*** 6104)	(***n =*** 96,275)	
**Multiplicity**	77,724 (75.9)			<.001^1^
Single	69,006	4177 (91.1)	64,829 (88.6)	
Two	5922	292 (6.4)	5630 (7.7)	
More than 3	2796	117 (2.6)	2679 (3.7)	
Missing	24,655	1518	23,137	
**Histologic grade**	101,082 (98.7)			<.001^1^
Low, intermediate	48,181	3057 (50.6)	45,124 (47.5)	
High	52,901	2988 (49.4)	49,913 (52.5)	
Missing	1297	59	1238	
**Histopathology**	102,379(100.0)			<.001^1^
In situ	10,402	453 (7.4)	9949 (10.3)	
IDC	75,924	4415 (72.3)	71,509 (74.3)	
ILC	2838	151 (2.5)	2687 (2.8)	
Mucinous carcinoma	1962	250 (4.1)	1712 (1.8)	
Others	11,253	835 (13.7)	10,418 (10.8)	
**Presence of lymphovascular invasion**	67,118 (65.6)			.006^1^
Yes	22,651	1288 (31.8)	21,363 (33.9)	
No	44,467	2768 (68.2)	41,699 (66.1)	
Missing	35,261	2048	33,213	
**ER**	90,827 (88.7)			.121^1^
Positive	61,811	3675 (69.0)	58,136 (68.0)	
Negative	29,016	1650 (31.0)	27,366 (32.0)	
Missing	11,552	779	10,773	
**PR**	90,732 (88.6)			<.001^1^
Positive	54,019	2964 (55.8)	51,055 (59.8)	
Negative	36,713	2346 (44.2)	34,367 (40.2)	
Missing	11,647	794	10,853	
**HER2**	76,508 (74.7)			<.001^1^
Positive	18,945	892 (19.6)	18,053 (25.1)	
Negative	57,563	3662 (80.4)	53,901 (74.9)	
Missing	25,871	1550	24,321	
**Ki-67**	45,789 (44.7)			<.001^1^
< 14%	22,213	1528 (51.9)	20,685 (48.3)	
≥ 14%	23,576	1418 (48.1)	22,158 (51.7)	
Missing	56,590	3158	53,432	
**Subtype**	76,349 (74.6)			
HR+/HER2- (Luminal A)	45,133	2841 (62.6)	42,292 (58.9)	<.001^1^
HR+/HER2+ (Luminal B)	9300	398 (8.8)	8902 (12.4)	<.001^1^
HR−/HER2+ (HER2)	9595	487 (10.7)	9108 (12.7)	<.001^1^
HR−/HER2- (TNBC)	12,321	813 (17.9)	11,508 (16.0)	<.001^1^
Missing	26,030	1565	24,465	
**pT**	98,583(96.3)			<.001^1^
T0, Tis, T1	60,484	3287 (56.1)	57,197 (61.7)	
T2, T3, T4	38,059	2572 (43.9)	35,487 (38.3)	
Tx	40	0	40	
Missing	0	0	0	
**pN**	98,583 (96.3)			.064^1^
N0, N1	87,344	5142 (87.9)	82,202 (88.7)	
N2, N3	11,175	707 (12.1)	10,468 (11.3)	
Nx	45	6	39	
Missing	19	4	15	
**pM**	98,583 (96.3)			.008^1^
M0	97,511	5775 (98.6)	91,736 (98.9)	
M1	1072	84 (1.4)	988 (1.1)	
Missing	0	0	0	
**p Stage**	98,583 (96.3)			<.001^1^
0, I	48,210	2667 (45.5)	45,543 (49.1)	
II, III, IV	50,373	3192 (54.5)	47,181 (50.9)	
Missing	0	0	0	
**Chemotherapy**	85,570 (83.6)			<.001^1^
Yes	56,714	1668 (34.4)	55,046 (68.2)	
No	28,856	3176 (65.6)	25,680 (31.8)	
Missing	16,809	1260	15,549	
**Chemotherapy**	56,714 (55.4)			<.001^1^
Neoadjuvant	2767	81 (5.1)	2686 (5.2)	
Adjuvant	49,519	1471 (91.8)	48,048 (92.2)	
Neoadjuvant and adjuvant	1032	23 (1.4)	1009 (1.9)	
Palliative	425	28 (1.7)	397 (0.8)	
Missing	2971	65	2906	
**Radiotherapy**	82,800 (80.9)			<.001^1^
Yes	51,950	1796 (37.8)	50,154 (64.3)	
No	30,850	2961 (62.2)	27,889 (35.7)	
Missing	19,579	1347	18,232	
**Radiotherapy**	51,950 (50.7)			.176^1^
Adjuvant	46,400	1642 (98.4)	44,758 (98.8)	
Palliative	590	27 (1.6)	563 (1.2)	
Missing	4960	127	4833	
**Hormonal therapy**	80,877 (79.0)			.937^1^
Yes	57,041	3416 (70.6)	53,625 (70.5)	
No	23,836	1424 (29.4)	22,412 (29.5)	
Missing	21,502	1264	20,238	

Values are either frequency with percentage in parentheses or median (range)

^1^ *P* values were derived from a Chi-square test

The Shapiro-Wilk test was employed to examine the normality assumption

*PR* Progesterone receptor, *ER* Estrogen receptor, *HER2* Human epidermal growth factor receptor 2

### Korean breast cancer occurence according to ages (Fig. 2)

The frequencies and incidence rates of total breast cancer, non-OBC, and OBC significantly increased with time (*p* < 0.001 for all). The frequency and incidence rates of OBC increased more radiply than did the non-OBC and total breast cancer (*p* < 0.001 for both frequency and incidence rate, from the regression analysis after Bonferroni correction). The proportion of OBC cases neared 4% in 2000. And this low proportion of OBC cases increased annually to 7.6% in 2013.

**Fig. 2:**
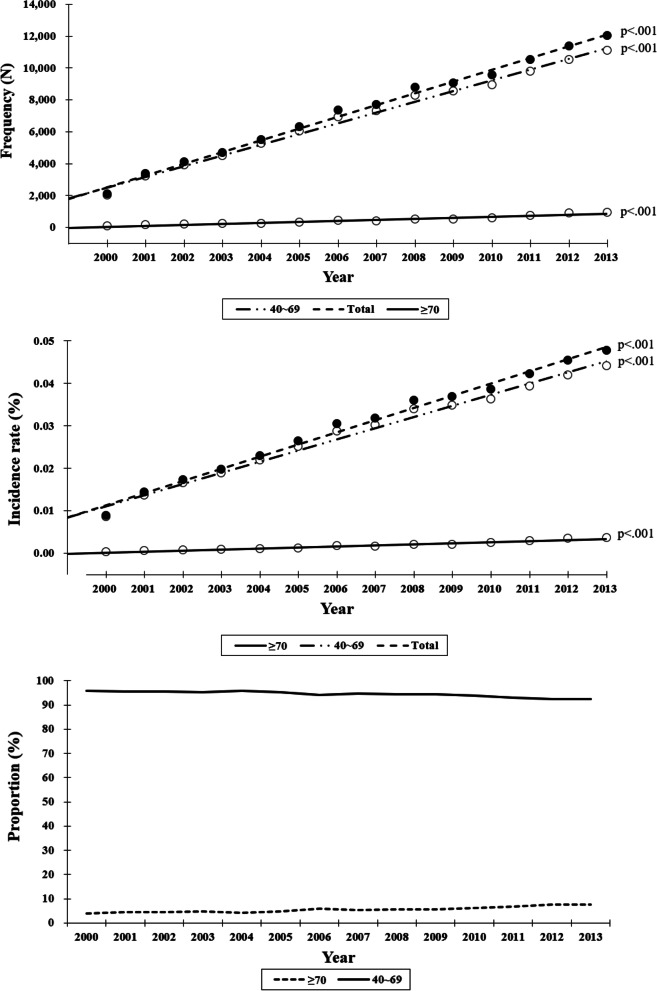
Breast cancer occurence with time (frequency, incidence rate, proportion) within age subgroups. The age subgroups that are compared are 40–69 and ≥ 70 years

### Korean breast cancer occurence according to the subtypes within different ages (Figs. 3, 4)

**Fig. 3:**
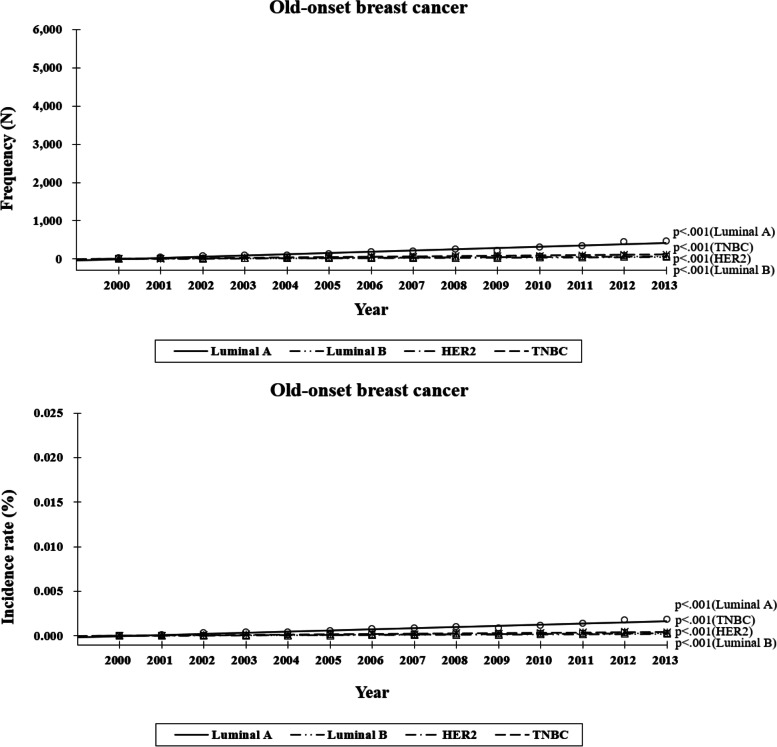
Breast cancer development (frequency, incidence rate) by subtype (luminal A, B, HER2, TNBC). Two age subgroups were compared (i.e., 40–69 and ≥ 70 years) among patients with old-onset breast cancer. HER, human epidermal growth factor receptor; TNBC, triple-negative breast cancer

We assessed how the frequency and incidence have changed with time according to the subtype within each age group. In the OBC group, both the frequency and incidence of all subtypes significantly increased with time (*p* < 0.001 for all, Fig. 3). However, regarding incidence, the rate of increase was significantly more higher in the luminal A subtype than did the remaining subtypes (*p* < 0.001). In descending order of numerical value, the frequencies of subtypes were estimated as 33.36 (luminal A), 8.25 (TNBC), 5.40 (HER2), and 4.31 (luminal B), respectively. In addition, their incidence were estimated as 0.000133 (luminal A), 0.000033(TNBC), 0.000021 (HER2), and 0.000017 (luminal B), respectively. Of all the subtypes, the incidence of the luminal A subtype increased the fastest, and the associated rate of increase was four to eight times higher than that of the other subtypes (*p* < 0.001).

**Fig. 4:**
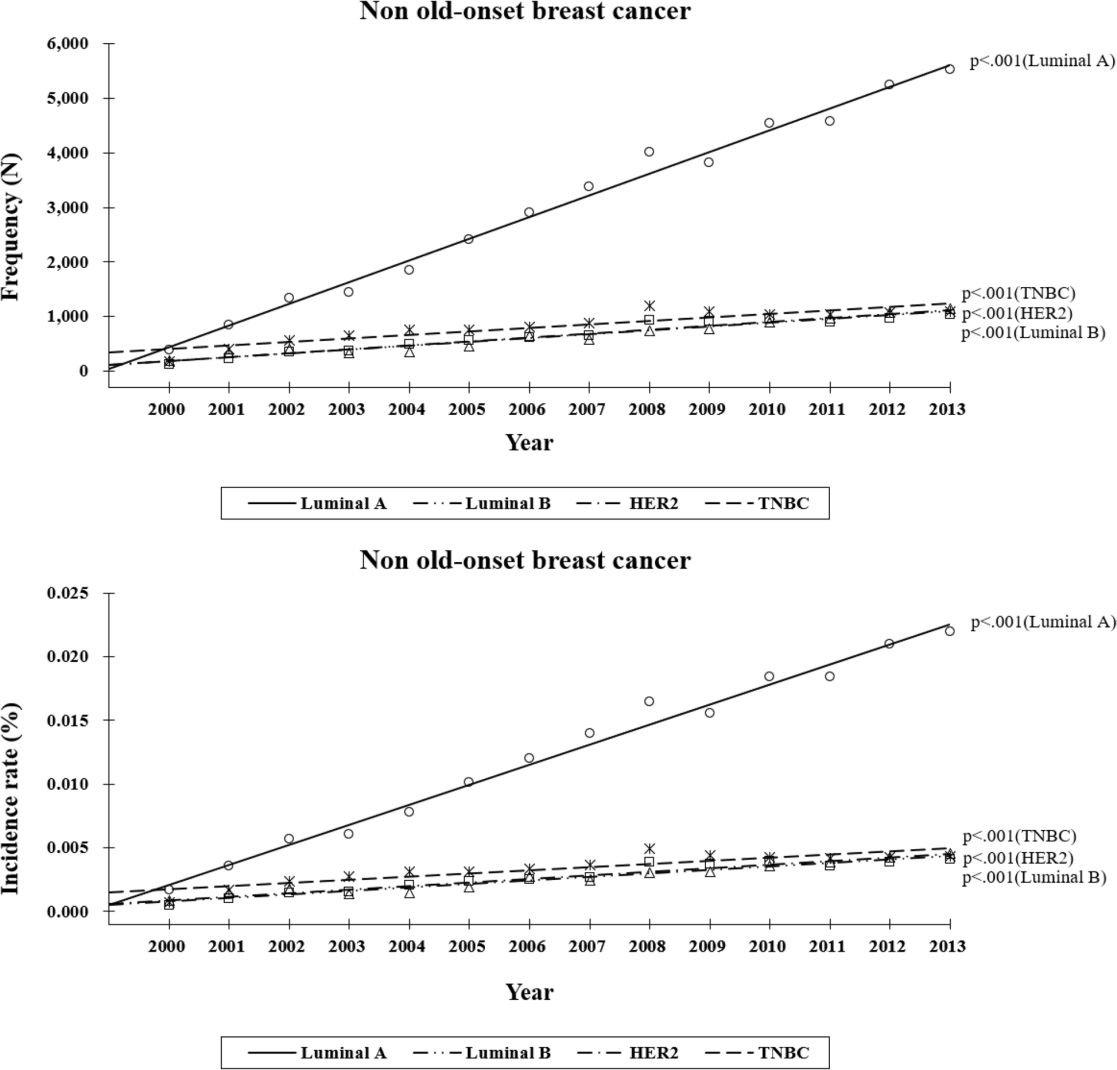
Breast cancer development (frequency, incidence rate) by subtype (luminal A, B, HER2, TNBC). Two age subgroups were compared (i.e., 40–69 and ≥ 70 years) among patients with non-old-onset breast cancer. HER, human epidermal growth factor receptor; TNBC, triple-negative breast cancer

In the non-OBC group, both the frequency and incidence of all the subtypes significantly increased with time (*p* < 0.001 for all, Fig. 4). In descending order of numerical value, the frequencies of subtypes were estimated as 397.45 (luminal A), 71.20 (HER2), 70.56 (luminal B), and 64.91 (TNBC), respectively. Moreover, the estimates of their incidence were 0.00157 (luminal A), 0.00028 (HER2), 0.00028 (luminal B), and 0.00025 (TNBC), respectively. Of all the subtypes, the luminal A subtype demonstrated the fastest increase in incidence, and the associated rate of increase was about six times higher than that of the other subtypes (*p* < 0.0001).

### Future trend in the proportion of OBC in Korea

Based on data from the KBCR, the porportion of OBC was low (6%) in Korea. However, the increase rate was steeper in OBC than non-OBC; consequently, the relative proportion of OBC has increased slowly over time. Assuming that the current trend persists, the relative proportion of OBC will eventually proceed toward the values reported in western countries.

## Discussion

This hospital-based database study showed that, in Korea, OBC has clinicopathological characteristics that are distinct from those of non-OBC, and that the incidences of total breast cancer, OBC, and non-OBC have continuously increased in the country. Noticeably, the increase in the incidence of OBC was slightly steeper than that of non-OBC in Korea; as a result, the proportion of OBC has been slowly increasing. The increase in the incidence of the luminal A subtype of breast cancer predominantly contributed to the increase in the incidence of both OBC and non-OBC, whereas increases in the incidence of other subtypes were relatively less evident.

In 2012, in the developed world, the ratio of female patinets with breast cancer at age ≥ 70 years accounted for 32.1% of all female patients with breast cancer at age ≥ 40 years [3]. Breast cancer incidence in Korea peaks at ages 45–49 years and, thereafter, declines with age [18]. In 2015, later-onset breast cancer at age ≥ 70 years account for only 8.8% of all breast cancer in Korea [17]. This pattern is unlike that noted in western countries, in which the incidence continuously increases with increasing age [8]. Although the proportion of OBC cases is still lower in Korea than in the West, our data show that the trend is shifting toward the values observed in the West, clearly indicating that age significanly increases the risk of breast cancer in the country. By 2050, the population of people who are 70 years or older is expected to rise sharply, up to 30.4% of the total population [19]. Considering the synergistic effect of the steep degree in population aging in Korea [19], the degree of increase in the relative ratio of OBC cases in the country may be larger than expected because the values noted in the West may be reached sooner than expected. The increase in the incidence of OBC can be attributed to prolonged life expectancy, which results in age-related biological alterations such as ER hypersensitivity, mammary epithelial cell changes, tumor microenvironment changes, and immune senescence that can increase one’s susceptibility to breast cancer [3].

In this study, OBC in Korea showed favorable biological features, except for the higher proportion of TNBC cases; however, there were discordantly advanced stages. OBC is considered to be different from non-OBC [3, 16]. Generally, OBC is characterized by favorable biological features, including a low tumor grade, low lympho-vascular invasion, histological types with good prognoses, more expressions of ER and PR, diploid and B-cell lymphoma 2 (Bcl-2) protein, and lesser expressions of HER2, Ki67, p53, and epidermal growth factor receptor [5, 14, 16, 20–30]. A recent meta-analysis focusing on OBC arrived at a parallel conclusion [3]. However, OBC is associated with a larger tumor size, greater lymph node involvement, a greater number of stage IV metastatic disease cases, and a more advanced overall tumor stage [3, 5, 14, 16, 25–27, 31–35]. Moreover, contrary to the general belief of the indolent course of OBC, growing evidence points to worse outcomes in older breast cancer patients [5, 33, 36]. The reasons for the worse outcomes in older patients can be attributed to under-treatment or variable disease biology, even within the same subtype [36]. Although breast cancer seemed to be more idolent in older patients than in younger ones, additional biological and genetic differences can exist even within similar disease subtypes [36].

Discrepancies between favorable biological features and advanced stages in OBC can be partly explained by an altered biological environment related to aging, which facilitates rapid disease progression, and also by society-related mechanisms such as the exclusion of elderly people from screening, or the notion of OBC being less aggressive, leading to diagnostic delays and the provision of proper care [3]. Along with omission of mammographic screening and self-breast examination, low perception of breast cancer in elderly women may also result in delayed diagnosis [26, 33].

Existing literature on OBC demonstrates great heterogeneities in terms of cancer stage, size, grade, and lymph node status at presentation, and clinical results in elderly women relative to their younger counterparts [3, 14, 16, 37]. The results of these parameters varied by study design and population, and either decreased or increased, or did not change with age [3, 14, 16, 37]. On average, elderly patients show a higher tumor stage than younger patients [14, 26, 33–35]. However, the proportion of those with an unknown stage increases with age because data on primary, nodal, and metastatic tumor statuses are frequently missed in elderly people [3, 14, 33, 34, 38]. Missing data in elderly populations can also be partly attributed to the discrepancy in terms of why breast cancer at older age demonstrate less aggressiveness in cancer biology despite its higher cancer stage at diagnosis, in contrast to breast cancer at younger age [14].

The distribution of breast cancer subtypes greatly fluctuates because of the complex effects of both age and race/ethnicity [39–45]. Regardless of race/ethnicity, the luminal subtype is universally the most predominant and is associated with the highest lifetime risk. Further, almost half of all luminal cancers develop after age 70 [40]. Breast cancer is biologically heterogeneous and intricate, and subtype alterations depending on age also vary with race [39]. A California-based study compared the proportions of breast cancer subtypes between between women groups at two different ages (40–69 years vs. ≥ 70 years), and evaluated how differently breast cancer subtypes varied according to age, depending on race [39]. The proportion of luminal A subtype cases increased after age 70 across people of all races, except Caucasians [39]. For White women, the proportion of the luminal A subtype was not influenced by age [39]. In Asian/Pacific Islander women, the proportion of the remaining subtypes other than luminal A did not change after age 70 [39]. Our data on native Korean women also show that the proportion of the luminal A subtype significantly after age 70. The global rise in the proportion of postmenopausal breast cancer and the luminal A subtype is regarded to be principally attributable to the global trend of acculturation to the Western lifestyle, including a high dietary consumption of animal fat, increased body mass index, and altered reproductive activities, such as nullipara, first childbirth at older age [17, 46–48]. For native Korean women, the increased incidence of the luminal A subtype was largely responsible for the overall increased incidence of both OBC and non-OBC, implying that environmental factors were predominantly responsible for the increased incidence of breast cancer in those aged 40 years and older.

In previous California-based studies, age did not significantly increase the risks of the TBNC and HER2 subtypes among Asian/Pacific Islander women [39, 49]. However, our data show that the proportion of all breast cancer subtypes did change after age 70. The proportion of the TNBC and luminal A subtypes significantly increased after age 70, whereas that of both the HER2+ and luminal B subtypes significantly decreased after that age. A recent study conducted in the United States showed that TNBC accounted for 8.4% of the 1,151,724 breast cancer cases [50]; this proportion varied according to race: 8.0% for non-Hispanic Whites, 18.1% for non-Hispanic Blacks, and 7.2% for Asians [50]. Compared with the proportion (7.2%) of TNBC observed in Asian-Americans [50], the proportions of the same in native Koreans (17.9% for OBC, 16.0% for non-OBC) are much higher, reaching the value (18.1%) observed in non-Hispanic Blacks. In the aforementioned population-based study, the proportion of TNBC cases decreased with age: 18.7, 10.5, 9.6, 7.7, 7.2% for ages < 40, 40–49, 50–64, 65–74, and ≥ 75 years, respectively), in accordance with the results of a California-based study [39]. However, interestingly, the proportion of TNBC cases in native Korean women increased after age 70. When considering the high proportion (23.8%) of TNBC cases among native Koreans under the age of 40 [17], the incidence of TNBC could have a bimodal peak before age 40 and after age 70 in native Korean women.

Our data, derived from the hospital-based voluntary cancer registry (KBCR), provide more specific information on patient and tumor characteristics, surpassing those derived from the nationwide obligatory cancer registry (KCCR). Nevertheless, the presence of selection bias cannot be ruled out. First, it cannot be guaranteed that KBCR data are fully representative of the actual breast cancer status in Korea. In fact, a period from 2000 to 2013, the proportions of OBC according to two major registry data were 6.0% (6104/102,379) in the KBCR, and 8.1% (11,162/137,540) in the nationwide cancer registry [51]. Table 3 summarizes annual difference in enrolled datasets between KBCR and KCCR. Indeed, in KBCR, enrolling institution and registered patients per each institution fluctuated annually because data collections were voluntarily carried out and altered by variable individual circumstances, which could influence results. However, our KBCR data can be somewhat representative of all Korean breast cancer cases. In terms of the differences in the proportions of OBC cases, as observed in the KBCR and nationwide registry, the 95% confidence interval of the difference was − 2.36 and − 1.95%, and the equivalence margin was 3%. Second, the matter of selection bias still persists for the subgroup analysis, as there were numerous cases with missing data on clinicopathological characteristics.

**Table 3:** Korean Breast Cancer Society’s registry relative to Korean central cancer registry datasets 2000–2013

Year	KBCS registry data (n)	KCCR data (n)	KBCR/KCCR (%)*
2000	2081	5848	35.6
2001	3368	7116	47.3
2002	4090	8033	50.9
2003	4698	8471	55.5
2004	5482	9179	59.7
2005	6316	10,228	61.8
2006	7354	10,900	67.5
2007	7701	11,988	64.2
2008	8783	12,786	68.7
2009	9062	13,631	66.5
2010	9539	14,627	65.2
2011	10,508	16,125	65.2
2012	11,375	16,717	68.0
2013	12,022	17,415	69.0

Values are presented as number or percentages*

Annual proportion of female patients with newly diagnosed breast cancer in:

*KCBS* Korean Breast Cancer Society Registry and *KCCR* Korea Central Cancer Registry

*Relative portion of numbers of dataset in KBCS to KCCR are presented as percentages

## Conclusions

In conclusion, based on a hospital-based database in Korea, although OBC in Korea was associated with favorable biological features, it showed a greater rate of the TNBC subtype and advanced disease stage. Currently, the Korean society continues to undergo popolution aging, and, accordingly, older Korean women ≥70 years old increasingly experienced breast cancer. Therefore, the management of breast cancer in this particular age group is expected to emerge as an urgent clinical issue in the near future. To lay the foundation for the management of OBC in Korea, there is a need for a comprehensive understanding of the disease’s characteristics, including the associated tumor biology and differences from non-OBC. Future studies should focus on this. Our results may provide novel insights into OBC in Korea, and aid in the development of optimal management of the disease in the country.

## Data Availability

The Korean Breast Cancer Registry, which was used for this study, is available with the permission of the Korean Breast Cancer Society. The corresponding author will provide information on where data supporting the results reported in this article can be found, including, where applicable, hyperlinks to publicly archived datasets analyzed or generated during the study.

## Abbreviations

DCIS: Ductal carcinoma in situ
ER: Estrogen receptor
FISH: Fluorescence in situ hybridization
HER: Human epidermal growth factor receptor
IDC: Invasive ductal carcinoma
IHC: Immunohistochemistry
ISH: In situ hybridization
KBCR: Korean Breast Cancer Registry
KBCS: Korean Breast Cancer Society
KCCR: Korean Central Cancer Registry
OBC: Old-onset breast cancer
PR: Progesterone receptor
SISH: Silver in situ hybridization
